# Protein kinase CK2α catalytic subunit is overexpressed and serves as an unfavorable prognostic marker in primary hepatocellular carcinoma

**DOI:** 10.18632/oncotarget.5470

**Published:** 2015-09-25

**Authors:** Hong-Xia Zhang, Shan-Shan Jiang, Xiao-Fei Zhang, Zi-Qi Zhou, Qiu-Zhong Pan, Chang-Long Chen, Jing-Jing Zhao, Yan Tang, Jian-Chuan Xia, De-Sheng Weng

**Affiliations:** ^1^ Sun Yat-Sen University Cancer Center, State Key Laboratory of Oncology in South China, Collaborative Innovation Center for Cancer Medicine, Guangzhou, China; ^2^ Department of Biotherapy, Sun Yat-Sen University Cancer Center, Guangzhou, China

**Keywords:** CK2α, hepatocellular carcinoma, prognosis, oncogene, apoptosis

## Abstract

Protein kinase CK2 alpha (CK2α), one isoform of the catalytic subunit of serine/threonine kinase CK2, has been indicated to participate in tumorigenesis of various malignancies. We conducted this study to investigate the biological significances of CK2α expression in hepatocellular carcinoma (HCC) development. Real-time quantitative polymerase and western blotting analyses revealed that CK2α expression was significantly increased at mRNA and protein levels in HCC tissues. Immunohistochemical analyses indicated that amplified expression of CK2α was highly correlated with poor prognosis. And functional analyses (cell proliferation and colony formation assays, cell migration and invasion assays, cell cycle and apoptosis assays) found that CK2α promoted cell proliferation, colony formation, migration and invasion, as well as inhibited apoptosis in hepatoma cell lines *in vitro*. CK2α-silenced resulted in significant apoptosis in cells that was demonstrated been associated with downregulation of expression of Bcl-2, *p*-AKT (ser473) and upregulation of expression of total P53, *p*-P53, Bax, caspase3 and cleaved-caspase3 in HCC cells. In addition, experiments with a mouse model revealed that the stimulative effect of CK2α on tumorigenesis in nude mice. Our results suggest that CK2α might play an oncogenic role in HCC, and therefore it could serve as a biomarker for prognostic and therapeutic applications in HCC.

## INTRODUCTION

Hepatocellular carcinoma (HCC, or hepatoma) is the most common primary malignancy of the liver in adults and the third leading cause of cancer-related deaths worldwide [1–3]. The highest incidence rates of HCC were reported in southeastern Asia and sub-Saharan Africa, occurring more often among men than women [4]. Although a significant improvement in survival was noted from the 1970s to 2000s, the rate of incidence has been increasing during the past few decades [5]. Although progresses in the epidemiology, etiology, biology, diagnosis and treatment and prolonging post-operative survival have been substantial, the long-term prognosis of patients with HCC remains poor [6, 7]. The potentially curative treatments for early-stage HCC include liver transplantation, hepatic surgical resection and early-stage radiofrequency ablation, but several factors limit the utility of these modalities. Noncurative treatment options for advanced HCC include new agents, such as sorafenib, systemic chemotherapy and transarterial chemoembolization [7, 8]. To a certain extent, these treatments have shown improvement in overall survival in early stage disease, but >70% of HCC patients who present with advanced disease would not benefit from them [3]. The crucial post-operative 5-year survival rate (30–40%) remains low and is an obstacle in the improvement of the prognosis for HCC patients [7]. As mechanisms of hepatocarcinogenesis are not completely understood, selecting novel molecular markers suitable for early diagnosis and new therapeutic targets to improve the outcome of patients with HCC is crucial.

Protein kinase CK2 (formerly casein kinase 2 or II) is a highly conserved and ubiquitous protein serine/threonine kinase. It has traditionally been classified as a messenger-independent protein kinase that consists of two catalytic subunits (42 kDa α, 38 kDa α’) and a regulatory subunit (28 kDa β) [9–12]. CK2 is a remarkably multifunctional protein kinase involved in the process of cell growth, proliferation and differentiation. And, subsequent studies have shown that CK2 is also a potent suppressor in the process of cell apoptosis [13–15]. The expression level of CK2α (catalytic subunit of protein kinase CK2) is well regulated in normal cells, but its aberrant expression and activity have been observed in many type of solid cancers, including lung, breast, prostate, gastric and kidney, as well as in hematopoietic malignancies, such as follicular, Burkitt and diffuse large B-cell lymphomas, acute myeloid leukemia and chronic lymphocytic leukaemia [16–24]. One study demonstrated that knockdown of CK2α resulted in obvious effects on cell proliferation, apoptosis, migration and cell cycle [25]. Furthermore, CK2 has been found to be involved in chromatin remodeling as well as protein transcription, translation and degradation [26–28]. The overexpression of CK2α has emerged as a poor prognosis marker for several cancers and a novel cancer therapeutic target. Such findings suggest that CK2α may have an oncogenic role in the development and progression of cancers. Even though CK2α has been investigated in various cancers, the detailed functional role of CK2α in human HCC has not been reported.

In the present study, we investigated the expression of CK2α in primary HCC and evaluated the prognostic value of CK2α for HCC patients. The biological function of CK2α in HCC progression was also explored using cell lines.

## RESULTS

### Overexpression of CK2α in human HCC

**Figure 1:**
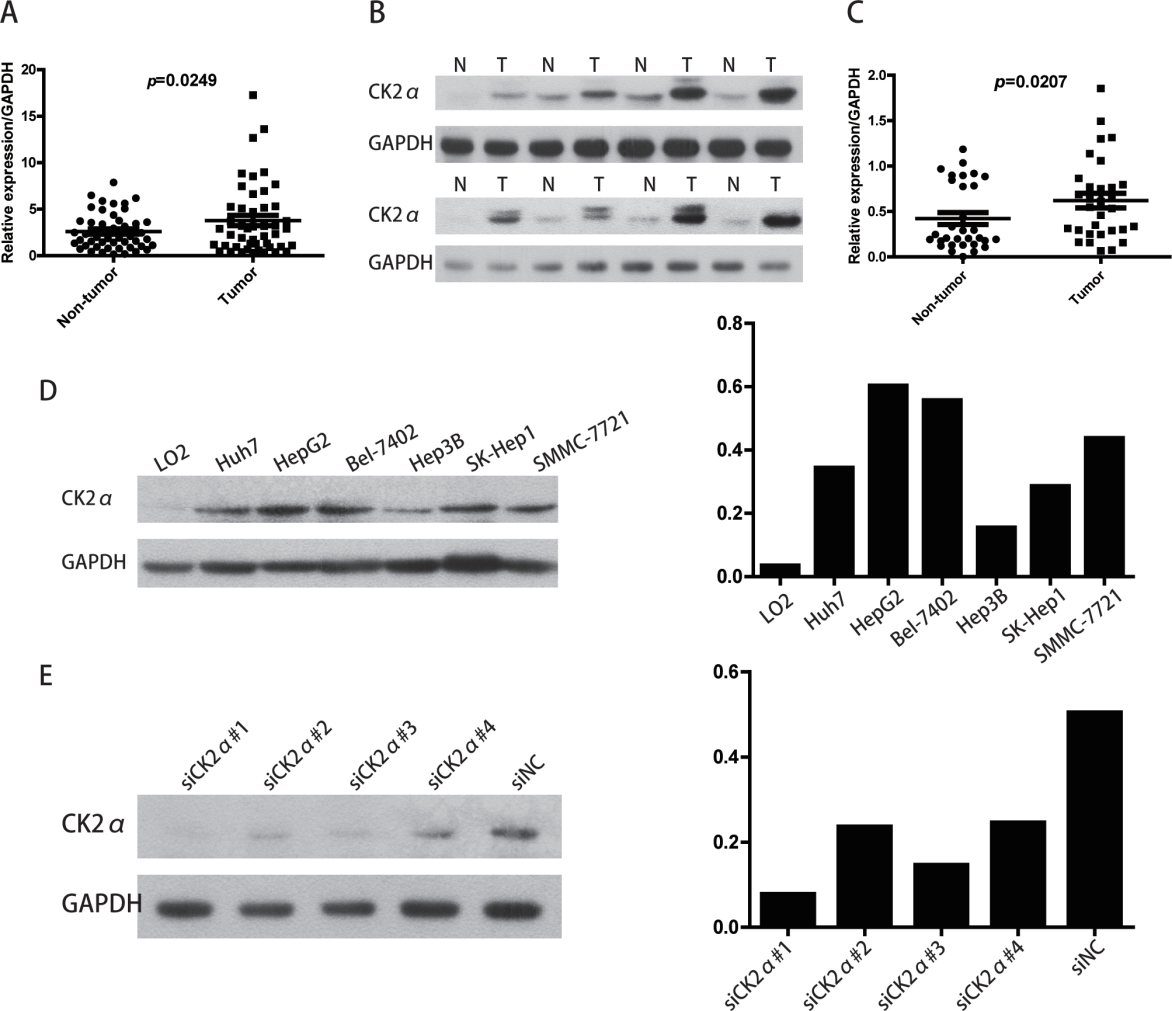
Expression level of CK2α mRNA and protein in human primary HCC cell lines and surgical specimens as evaluated by RT-qPCR and Western blot **A.** RT-qPCR showed that relative *CK2α* mRNA expression was higher in 47 HCC tissues than in matched adjacent non-cancerous tissues (*P* = 0.0249). **B.** Representative Western blot results showed that the expression of CK2α protein in eight HCC tissues was significantly higher than that in matched adjacent non-cancerous tissues (N, non-tumor, T, tumor). **C.** Relative expression of CK2α protein was increased remarkably in 31 HCC tissues compared with matched adjacent non-cancerous tissues (*P* = 0.0207). **D.** CK2α protein was up-regulated in Huh7, HepG2, BEL-7402, Hep3B, SK-Hep1 and SMMC-7721 cells (particularly in Bel-7402 and HepG2 cells) compared with the normal liver cell line LO2. **E.** Among the four tested siRNAs against CK2α, siCK2α#1 and siCK2α#3 showed higher knockdown efficiencies.

Primary paired HCC tissue samples and HCC cell lines were used to examine CK2α expression. The *CK2α* gene expression was analyzed by RT-qPCR in 47 pairs of HCC tissues and their corresponding non-tumorous liver tissues. Compared with corresponding non-tumorous liver tissues, *CK2α* at the mRNA level was significantly and frequently (63.8%, 30/47) overexpressed (defined as a greater than two-fold increase) in the HCC tissues (*P* = 0.0249, paired Student's *t*-test; Figure 1A). To investigate whether differences in expression in the mRNA level would be reflected at the protein level, Western blot analysis was conducted. Consistent with the RT-qPCR results, CK2α protein expression was significantly higher in the HCC tissues compared to non-tumor tissues (70.9%, 22/21, *P* = 0.0207, paired Student's *t*-test; Figure 1B, 1C). By Western blot analysis, protein levels of CK2α in all six HCC cell lines evaluated (Huh7, Bel-7402, HepG2, Hep3B, SK-Hep1, SMMC-7721) were found to be increased (particularly in Bel-7402 and HepG2 cells), compared with the normal liver cell line LO2 (Figure 1D). Thus, CK2α expression was positively associated with HCC progression, suggesting that it plays an oncogenic role in HCC.

### Immunohistochemical (IHC) analysis of CK2α expression in HCC clinical samples and its relationship with patient survival

**Figure 2:**
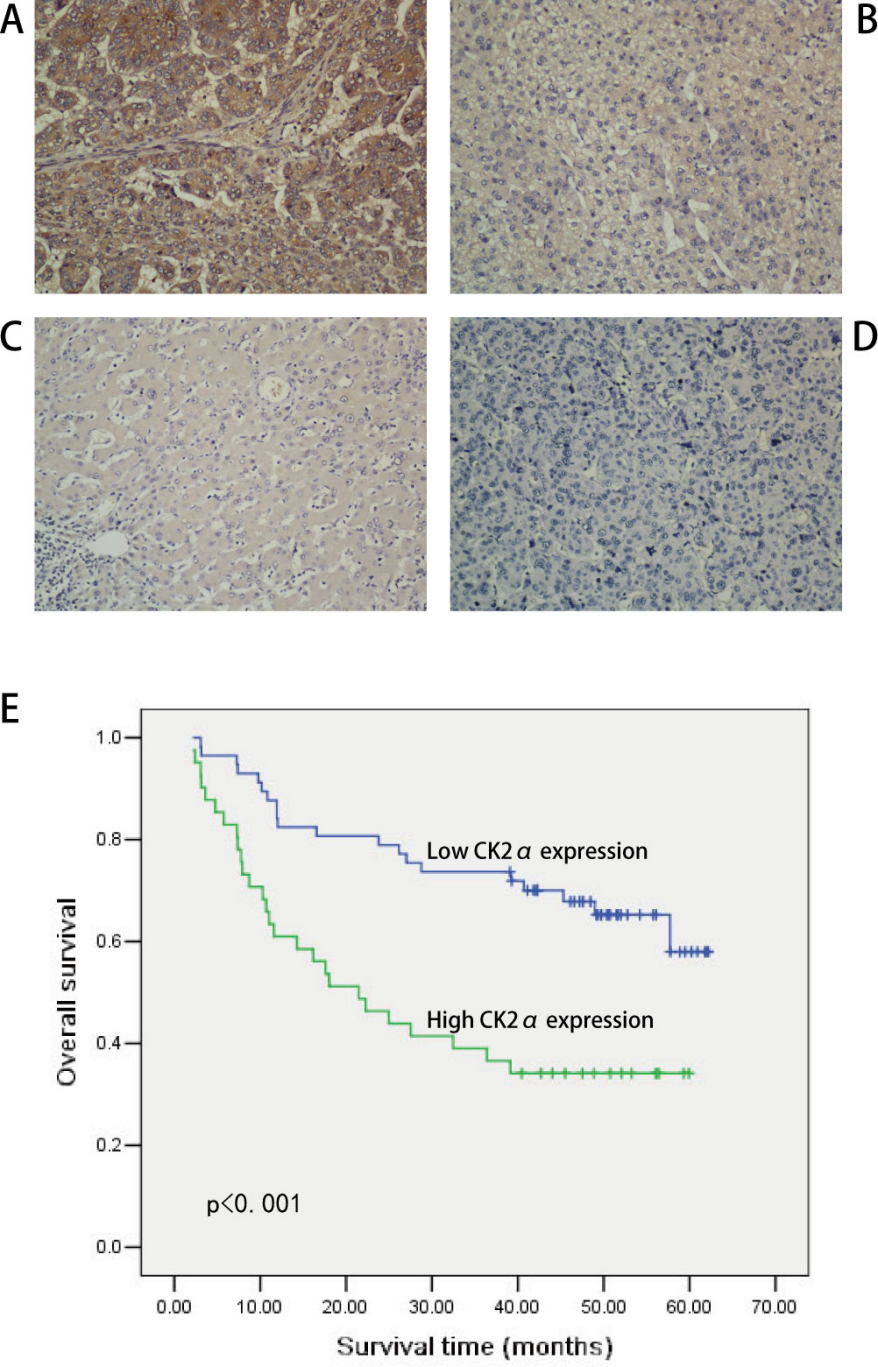
IHC analyses of CK2α protein expression in primary HCC surgical specimens and Kaplan–Meier survival analyses of the primary HCC patients (*n* = 98) **A.** Strong CK2α staining in HCC, scored as CK2α (+++). **B.** Moderate CK2α staining in HCC, scored as CK2α (++). **C.** Weak CK2α staining in HCC, scored as CK2α (+). **D.** CK2α-negative staining in HCC, scored as CK2α (−). All images are shown at × 200 magnification. **E.** Based on CK2α immunostaining analysis of their tumors, HCC patients were divided into low-CK2α expression (*n* = 56, CK2α- or CK2α+) and high-CK2α expression (*n* = 42, CK2α++ or CK2α+++) groups. Survival of patients in the low-CK2α group was significantly higher than that of patients in the high-CK2α group (*P* < 0.001, log-rank test).

To further explore the role and prognostic value of CK2α in human HCC, 98 paraffin-embedded primary HCC samples confirmed by histopathology were used to examine CK2α expression using IHC. In the CK2α-positive specimens, CK2α was detected in the cytoplasm and cell membrane (Figure 2A–2C). CK2α expression was negative in non-tumorous liver parenchyma (Figure 2D). High CK2α expression (++ or +++) was found in 42 (42.9%) specimens, and low CK2α expression (− or +) was detected in 56 (57.1%) specimens (Table 1). Correlations between the clinicopathological parameters of HCC and expression of CK2α are summarized in Table 1. Chi-square analyses revealed that CK2α expression was positively correlated with histological grade (*P* = 0.033), distant metastasis (*P* = 0.003) and tumor stage (TNM) (*P* = 0.012), but not with tumor size, liver cirrhosis, vascular invasion, serum AFP or tumor capsule (Table 1). Kaplan-Meier analyses revealed a significant association between high CK2α expression and poor prognosis (*P* < 0.001, Figure 2E). Overall survival was significantly higher in the group with low CK2α expression than that in the group with high CK2α expression. Further univariate and multivariate analyses were employed to compare the associations of CK2α expression with other clinicopathological parameters. Univariate Cox regression analyses showed that CK2α expression (*P* = 0.001), histological grade (*P* = 0.015), distant metastasis (*P* = 0.001) and tumor stage (TNM) (*P* = 0.002) were significant risk factors (Table 2). Multivariate Cox regression analyses indicated CK2α expression as an independent prognostic factor (*P* = 0.030, Table 2). Therefore, CK2α may be an important marker for predicting the overall survival of HCC patients.

**Table 1:** Correlation between CK2α expression and clinicopathological variables of 98 patients with HCC

Clinicopathologic variable	CK2α expression			χ^2^	*P*
	*N*	Low	High		
All cases	98	56	42		
Age (years)				3.16	0.075
<50	51	34	17		
≥50	47	23	24		
Sex				0.015	0.901
Male	88	51	37		
Female	10	6	4		
Tumor size(cm)				1.478	0.224
<5	33	22	11		
≥5	65	35	30		
Histological grade				9.453	0.009^a^
Good	37	28	9		
Moderate	28	16	12		
Poor	33	13	20		
Liver cirrhosis				1.781	0.182
No	26	18	8		
Yes	72	39	33		
HBV				0.115	0.735
Negative	13	7	6		
Positive	85	50	35		
AFP (ng/mL)				1.349	0.245
Negative (≤400)	53	28	25		
Positive (>400)	45	29	16		
Tumor capsule				1.087	0.297
Intact	32	21	11		
Absent and not intact	66	36	30		
Vascular				0.004	0.952
No	81	47	34		
Yes	17	10	7		
TNM stage)				7.365	0.007^a^
Stage I	54	38	16		
Stage II and III	44	19	25		
Distant metastasis				4.436	0.035^a^
No	77	49	28		
Yes	21	8	13		

AFP, alfa fetoprotein; HBV, hepatitis B virus.

a *P* < 0.05.

**Table 2:** Univariate and multivariate analyses of overall survival in HCC patients

Variable	Univariate analyses			Multivariate analyses		
	HR	95%CI	*P*	HR	95%CI	*P*
CK2α	2.708	1.512–4.850	0.001^a^	1.971	1.006–3.642	0.03^a^
Age	1.148	0.647–2.036	0.637			
Sex	0.767	0.275–2.137	0.612			
Tumor size	1.790	0.929–3.450	0.082			
Histological grade	1.520	1.084–2.132	0.015^a^			0.849
Liver cirrhosis	1.557	0.774–3.132	0.215			
HBV	0.922	0.413–2.059	0.843			
AFP	1.733	0.975–3.078	0.061			
Tumor capsule	0.576	0.322–1.030	0.063			
Vascular	1.837	0.930–3.626	0.080			
TNM stage	2.535	1.411–4.554	0.002^a^	2.008	1.094–3.686	0.024^a^
Distant metastasis	2.666	1.463–4.858	0.001^a^	2.120	1.144–3.926	0.017^a^

HR, hazard ratio; CI, confidence interval; AFP, alfa fetoprotein; TNM, tumor, node, metastasis.

a *P* < 0.05.

### Inhibition of CK2α expression in HCC cell lines

Western blot analysis showed relatively higher expression of CK2α in Bel-7402 and HepG2 cells than the other cell lines tested (Figure 1D). Accordingly, we selected Bel-7402 and HepG2 as the optimal cells to transfect with four CK2α-targeting siRNAs (siCK2α#1, siCK2α#2, siCK2α#3, siCK2α#4) in order to investigate the biological function of CK2α in HCC cell lines. The knockdown effect of CK2α was evaluated by Western blot analysis. We noted that CK2α expression levels were markedly decreased in cells transfected with siCK2α#1 and siCK2α#3 when compared with those treated with siCK2α#2 and siCK2α#4 (Figure 1E).

### CK2α promotes hepatoma cell proliferation

**Figure 3:**
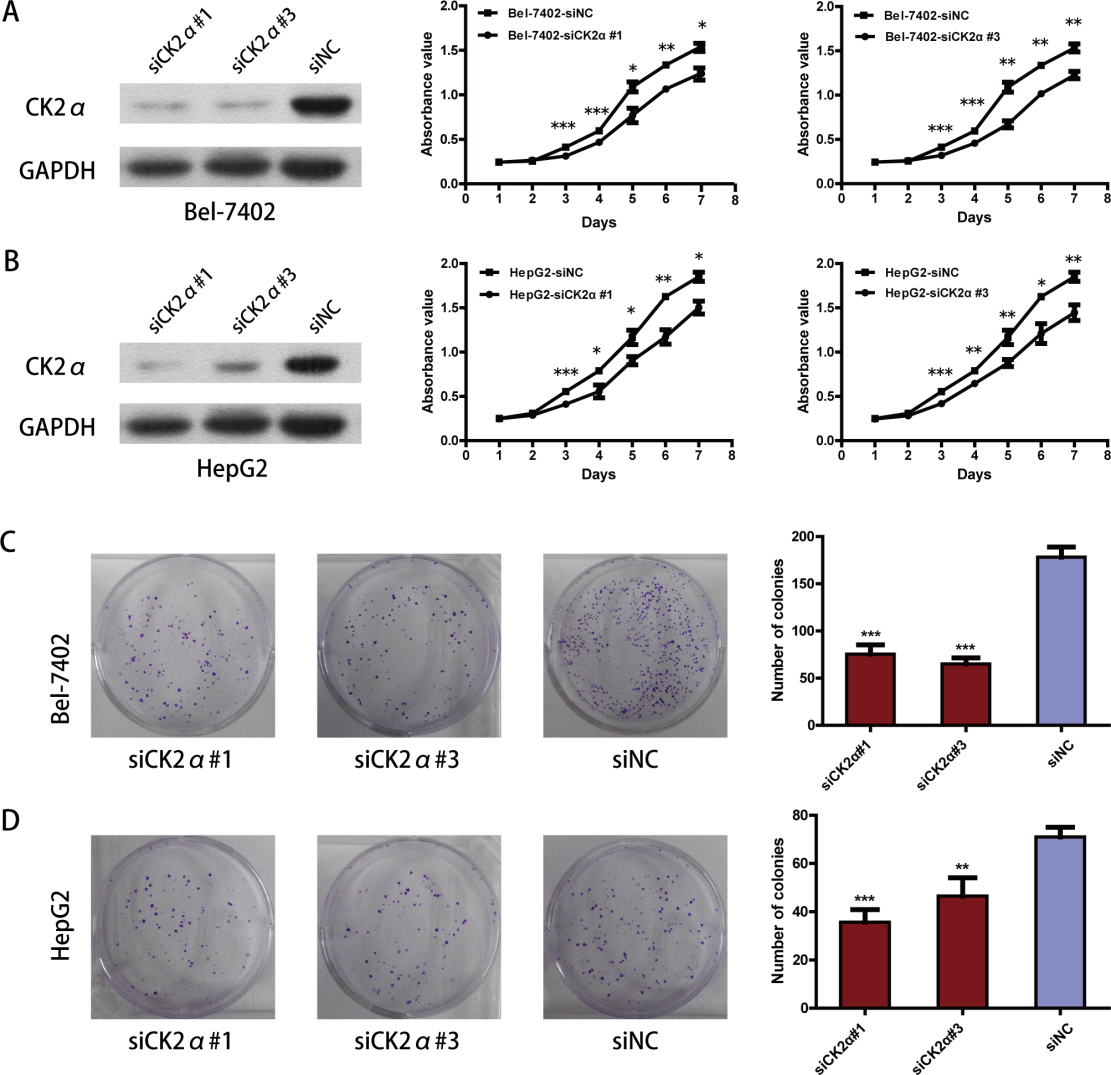
CK2α was essential for hepatoma cell proliferation and colony-formation **A, B.** Knockdown efficiency of selected CK2α-targeting siRNAs in transfected cells was evaluated by Western blot, and the MTS assay showed that silencing of CK2α suppressed proliferation of Bel-7402 (A) and HepG2 (B) cell lines. **C, D.** Colony-formation assays indicated decreased growth rates in CK2α-silenced Bel-7402 (C) and HepG2 (D) cell lines. All images are shown at ×200 magnification. Experiments were carried out in triplicate. Data are presented as the mean ± SD of three independent experiments. *P*-values were calculated using the independent Student's *t*-test. *P < 0.05 versus control; **P < 0.01 versus control; ***P < 0.001 versus control.

**Figure 4:**
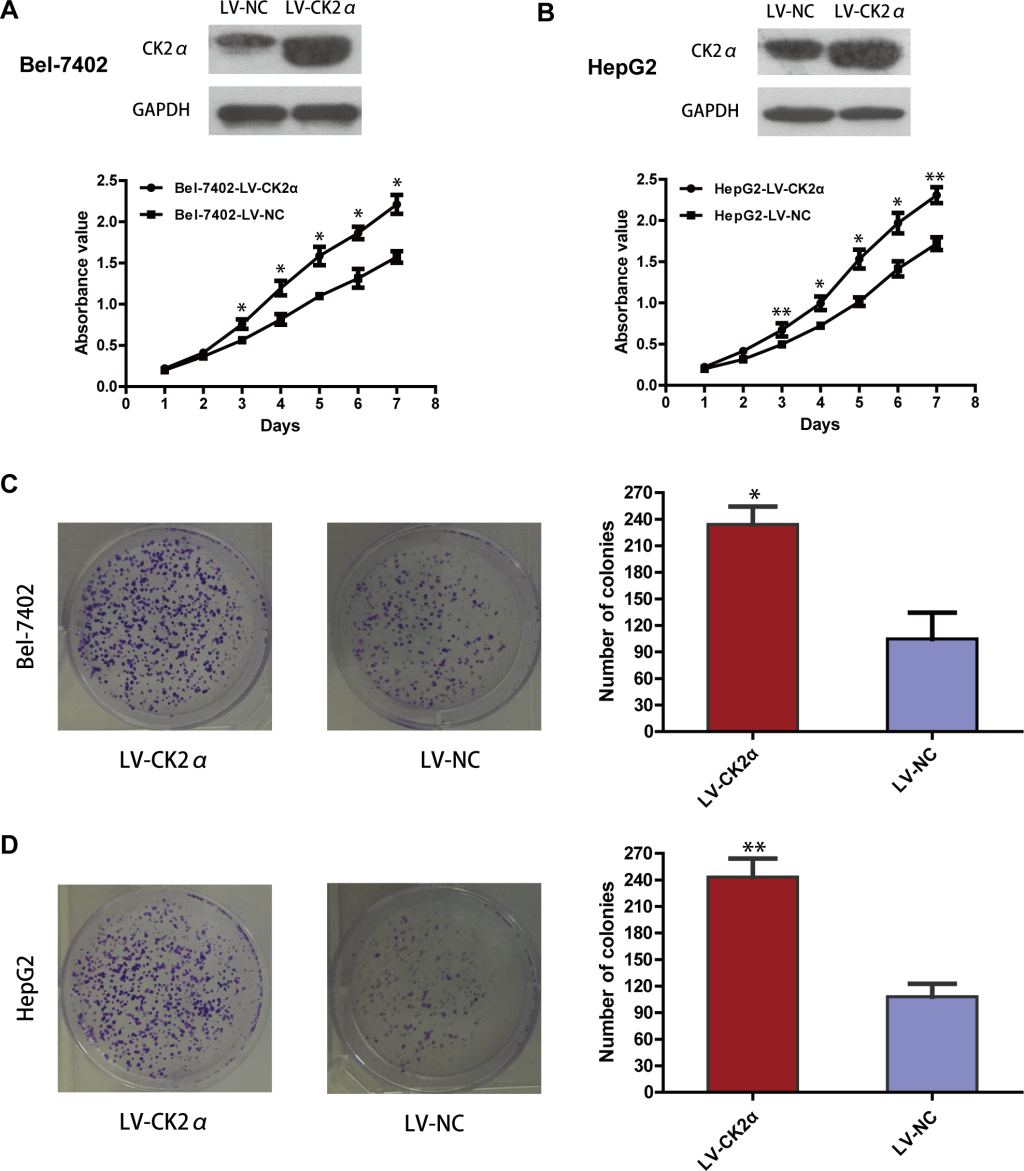
Growth-promoting role of CK2α in Bel-7402 and HepG2 cell lines **A, B.** Overexpressed efficiency of selected CK2α recombinant lentiviral vector in transfected cells was evaluated by Western blot, and the MTS assay showed that overexpressing of CK2α promote proliferation of Bel-7402 (A) and HepG2 (B) cell lines. **C, D.** Colony-formation assays indicated increased growth rates in CK2α-overexpressed Bel-7402 (C) and HepG2 (D) cell lines. All images are shown at ×200 magnification. Experiments were carried out in triplicate. Data are presented as the mean ± SD of three independent experiments. *P*-values were calculated using the independent Student's *t*-test. *P < 0.05 versus control; **P < 0.01 versus control.

We carried out cell proliferation and colony formation assay to explore the role of CK2α in the growth of hepatoma cells. After Bel-7402 and HepG2 cells were transiently transfected with CK2α-specific siRNAs and siNC RNA (NC, negative control) for 48 h, they were evaluated in cell proliferation assays and colony-forming assays. Cell proliferation (*P* < 0.05, Figure 3A, 3B) as well as colony-formation abilities (*P* < 0.01, Figure 3C, 3D) were significantly inhibited in Bel-7402 and HepG2 cells transiently transfected with siCK2α compared with those transfected with siNC. Whereas the Bel-7402 and HepG2 cells infected with LV-CK2α showed increased growth rates (*P* < 0.05, Figure 4A, 4B) and greater colony-forming abilities (*P* < 0.05, Figure 4C, 4D). These results further supported that CK2α promote the growth of HCC cells in hepatocarcinogenesis.

### CK2α promotes migration and invasion of HCC cells *in vitro*

**Figure 5:**
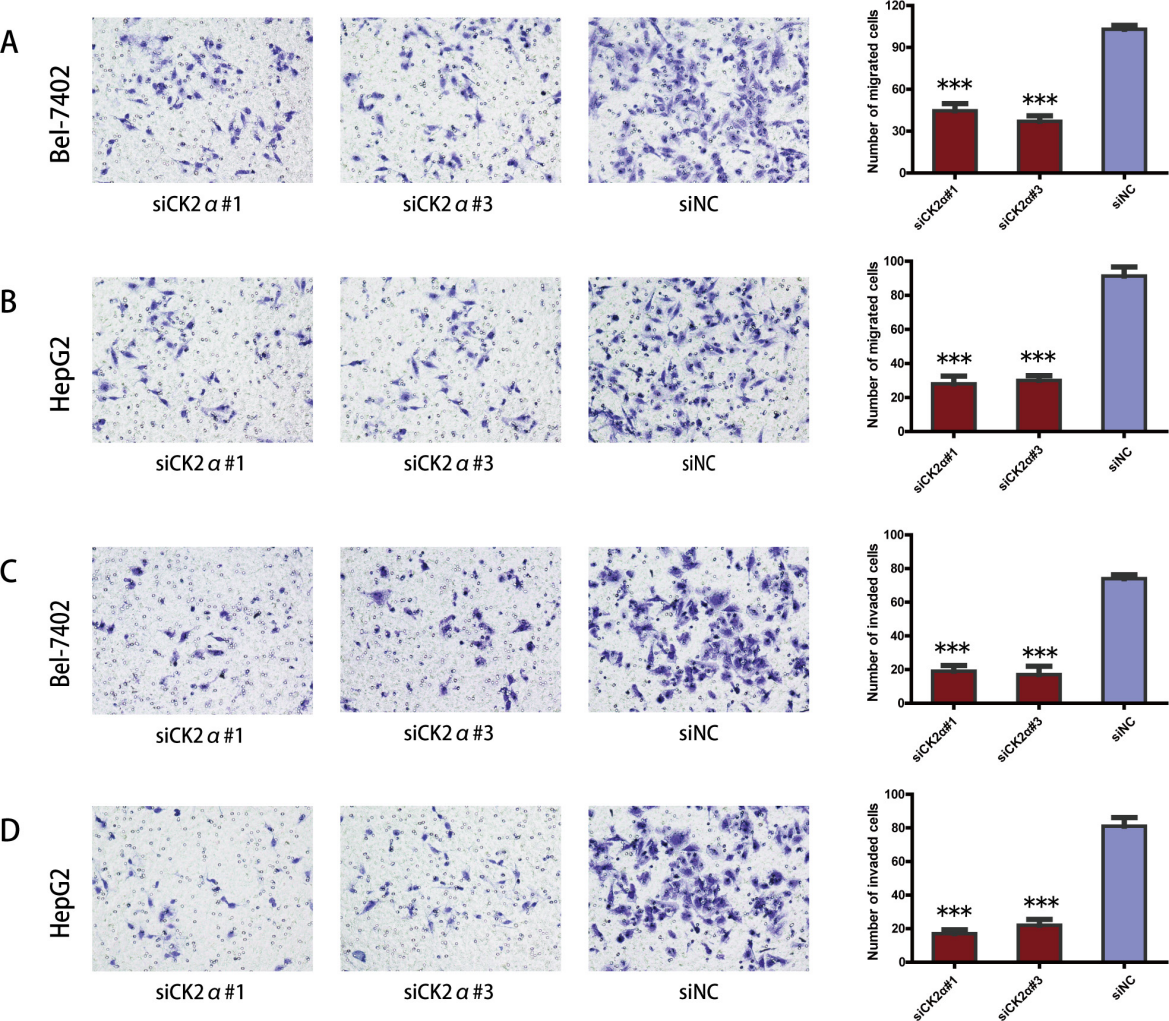
Suppression of hepatoma cell migration and invasion ability by CK2α silencing **A, B.** CK2α knockdown using specific siRNAs inhibited the migration ability of Bel-7402 (A) and HepG2 (B) cells in a Transwell migration assay. **C, D.** CK2α silencing using specific siRNAs remarkably attenuated the invasion ability of Bel-7402 (C) and HepG2 (D) cells in a Matrigel invasion assay. All images are shown at ×200 magnification. Data are presented as the mean ± SD of three independent experiments. *P*-values were calculated using the independent Student's *t*-test. ***P < 0.001 versus control.

**Figure 6:**
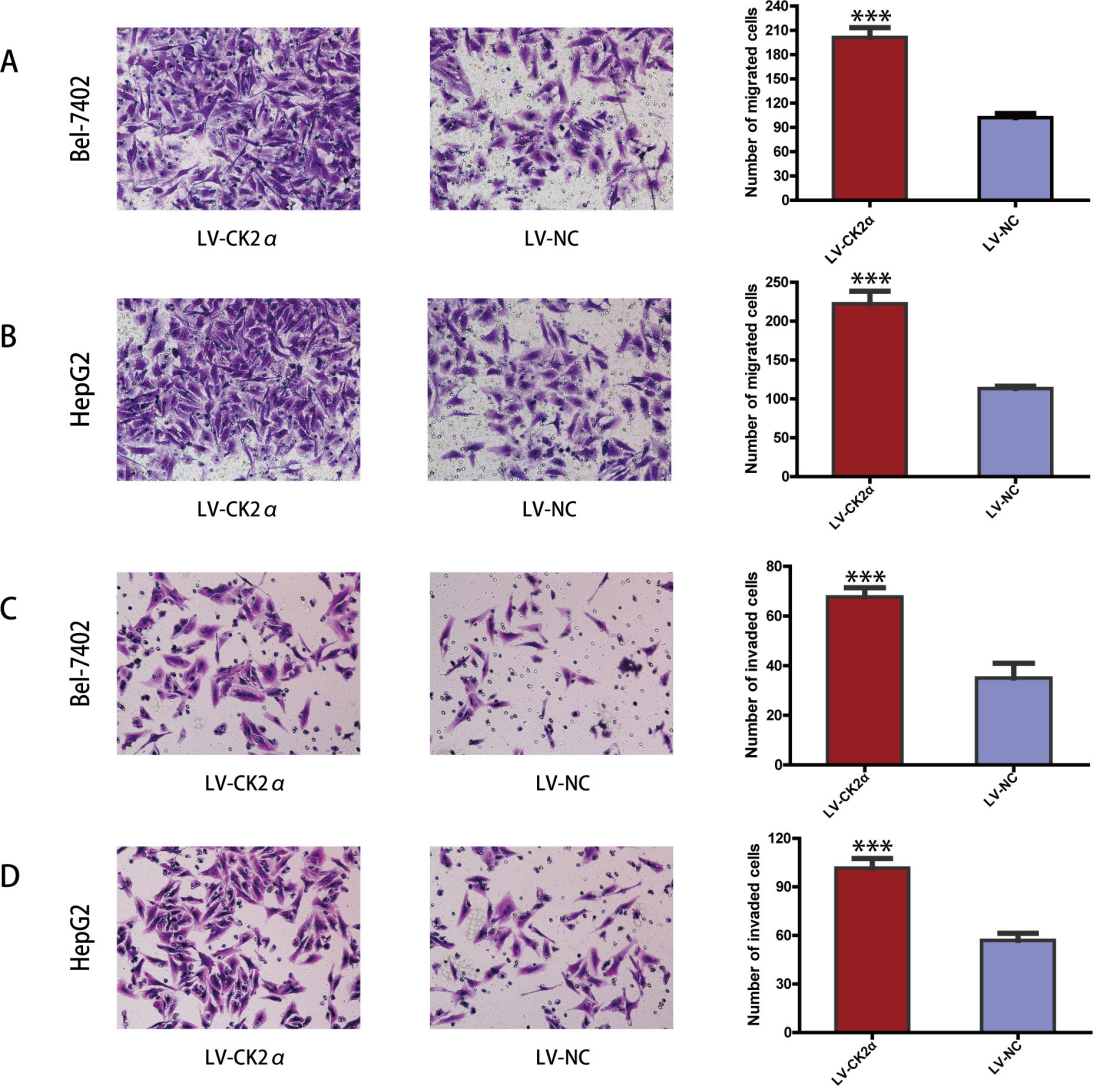
CK2α overexpression promoted hepatoma cell migration and invasion **A, B.** CK2α overexpression using specific recombinant lentiviral vector promote the migration ability of Bel-7402 (A) and HepG2 (B) cells in a Transwell migration assay. **C, D.** CK2α overexpression using specific recombinant lentiviral vector remarkably increase the invasion ability of Bel-7402 (C) and HepG2 (D) cells in a Matrigel invasion assay. All images are shown at ×200 magnification. Data are presented as the mean ± SD of three independent experiments. *P*-values were calculated using the independent Student's *t*-test. ***P < 0.001 versus control.

The previous data revealed that the upregulation of CK2α expression was significantly associated with advanced clinical stages (Table 1). Thus, we undertook further *in vitro* studies using the transwell migration assay to examine the effect of CK2α on hepatoma cell motility. Transient transfection of Bel-7402 and HepG2 cells with siCK2α led to significantly suppressed cell migration and invasion through the membrane in the chamber as compared with control cells (Figure 5A, 5B, 5C, 5D). And the ability of migration and invasion in Bel-7402 and HepG2 cells infected with LV-CK2α was significantly increased (Figure 6A, 6B, 6C, 6D). Together, these results provide evidence that upregulated CK2α expression levels are important for the aggressive characteristics of HCC cells.

### CK2α silencing induces apoptosis in HCC cell lines

**Figure 7:**
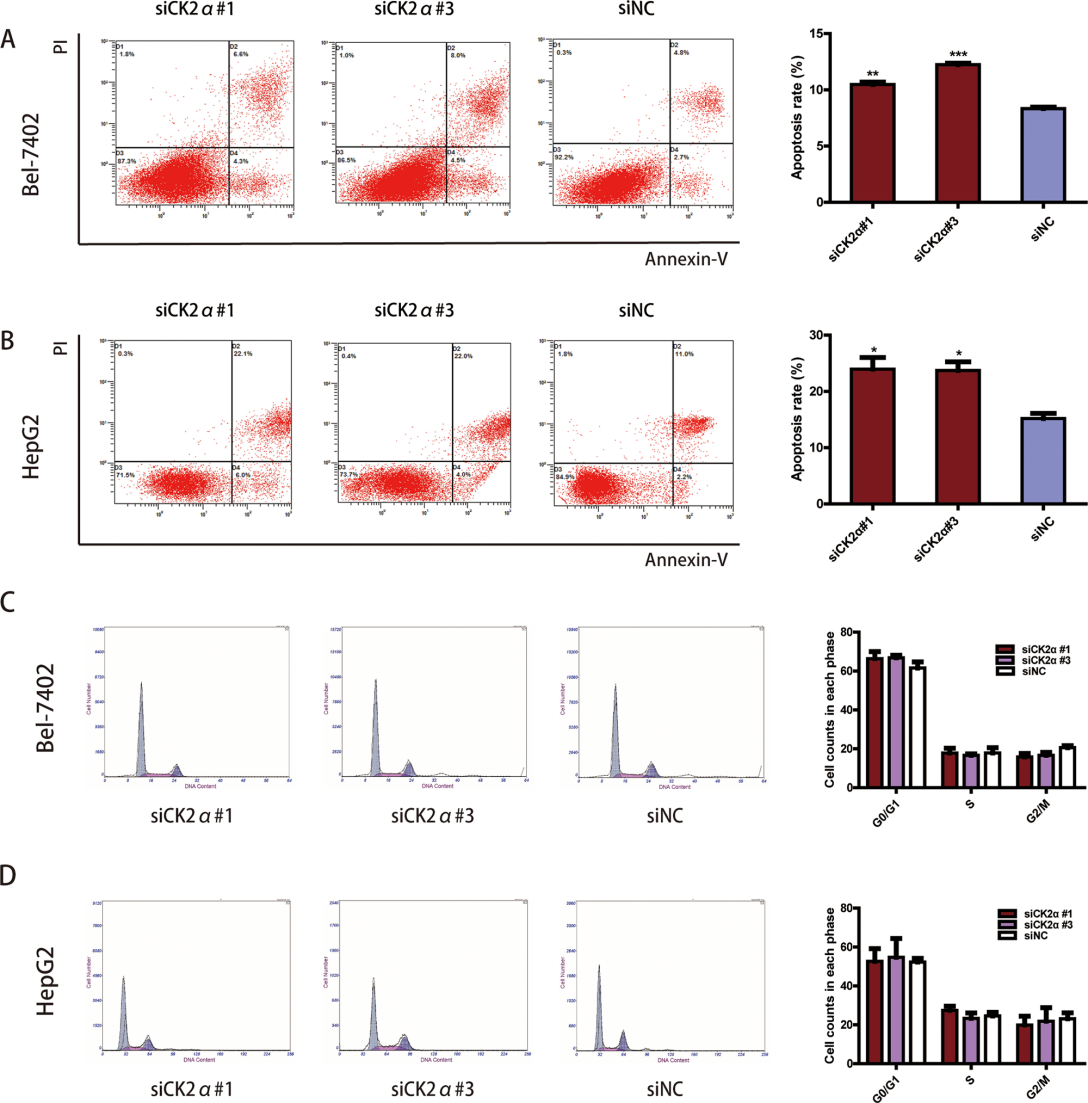
Effect of CK2α on apoptosis and cell cycle in HCCs **A, B.** CK2α silencing by siRNAs of Bel-7402 (A) and HepG2 (B) cells significantly increased cell apoptosis. *P < 0.05 versus control; **P < 0.01 versus control; ***P < 0.001 versus control. **C, D.** Cell-cycle distribution was not changed significantly between Bel-7402 (C) and HepG2 (D) cells transfected with CK2α-specific siRNAs and siNC. *P < 0.05 versus control. Data are presented as the mean ± SD of three independent experiments.

To explore whether the CK2α knockdown-mediated suppression of cell growth is associated with cell cycle arrest or an induction of apoptosis, we performed cell cycle and apoptosis analyses using flow cytometry. Significant differences in Annexin V-positive apoptotic cells based on flow cytometry were observed in the CK2α siRNAs treated groups in comparison to cells transfected with siNC. Apoptosis was induced in 10.47 ± 0.40% and 12.23 ± 0.23% of the Bel-7402 cells transfected with siCK2α#1 and siCK2α#3, respectively, compared with 8.33 ± 0.21% of those treated with siNC (*P* < 0.001, Figure 7A). Similarly, siCK2α#1 and siCK2α#3 induced apoptosis in 23.97 ± 3.58% and 23.70 ± 2.71% of HepG2 cells, respectively, compared with 15.20 ± 1.57% of those treated with siNC (*P* < 0.05, Figure 7B). Cell cycle analysis indicated that the proportions of cells distributed in G0/G1, S and G2/M phases were not significantly changed in Bel-7402 (*P* > 0.5 for siCK2α#1 and siCK2α#3, Figure 7C) or HepG2 (*P* > 0.5 for siCK2α#1 and siCK2α#3, Figure 7D) cells transfected with CK2α siRNAs compared with those transfected with siNC. These results suggested that CK2α may promote HCC development through anti-apoptotic process.

### The regulatory mechanism of CK2α on cell apoptosis

**Figure 8:**
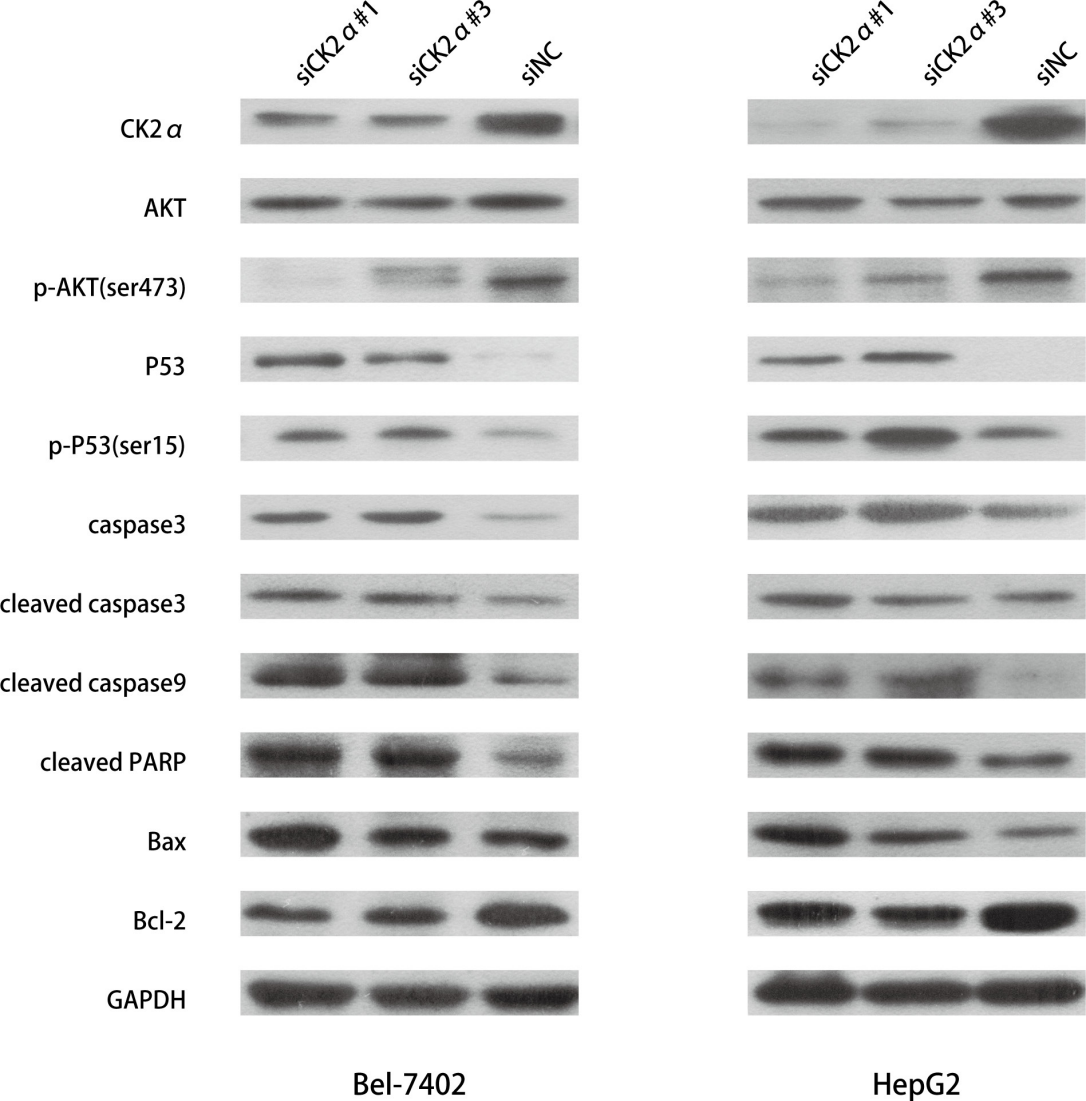
Detection of apoptosis-related proteins by western blotting Downregulation of expression of *p*-AKT (ser473) and Bcl-2 were detected in CK2α-knockdown Bel-7402 and HepG2 cells. The expression of P53, *p*-P53 (ser15), caspase3, cleaved caspase3, cleaved caspase9, cleaved PARP, Bax were increased in CK2α-knockdown Bel-7402 and HepG2 cells.

The apoptosis assay indicated that CK2α plays anti-apoptotic role in HCC development. To investigate the regulatory mechanism of CK2α on cell apoptosis, we conducted western blotting to detect apoptosis related proteins. Caspases are central components in the induction of apoptosis, of which, caspase3 is a crucial executioner of cell apoptosis [29]. We measured the change in the expression of caspase3 and cleaved caspase3 when CK2α was silenced in HCC cells. And we also detect cleaved caspase9 and cleaved PARP. In accord with the apoptosis assay indicating, we found that the protein levels of caspase3, cleaved caspase3, cleaved caspase9 and cleaved PARP were strongly increased in HCC cells treated with siCK2α as compared with the siNC group (Figure 8). In previous study, Matthew S. Brown *et al.* found that CK2α play a critical role in anti-apoptosis through negatively regulating the level of *TP53* family protein [25]. P53 plays an important role in the apoptosis of mitochondrial-dependent pathway. In our study, we assessed the effect of CK2α knockdown on regulation of P53 status protein by western blot. Our results indicated that CK2α knockdown increased total P53 and phosphorylation P53 in HCC cell lines (Figure 8). As the downstream mediators of P53-dependent apoptosis, Bcl-2 family proteins are key regulators of the apoptotic pathway [30]. The Bcl-2 family includes the anti-apoptotic protein Bcl-2 and pro-apoptotic protein Bax. In our study, we found that the expression of Bcl-2 was down-regulated and Bax up-regulated when CK2α was silenced in HCC cell lines (Figure 8). The expression of Bcl-2/Bax was down-regulated in CK2α-knockdown Bel-7402 and HepG2 cells. And previous study indicated that the PI3K/AKT signaling pathway plays a significantly role in regulating cell survival and apoptosis [31]. We measured the level of AKT and *p*-AKT (ser473) in HCC cells conducted by siCK2α and siNC. The results showed that *p*-AKT (ser473) decreased in cells treated with siCK2α (Figure 8). All these results indicated that CK2α plays anti-apoptosis role in HCC development.

### CK2α promotes tumorigenesis of HCC *in vivo*

**Figure 9:**
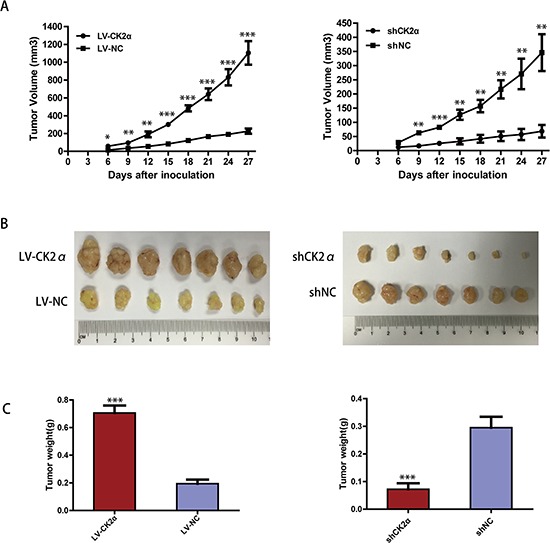
CK2α increase the tumorigenicity of HCC *in vivo* **A.** The tumor growth curves for each group. The tumor growth rate was elevated in the tumors that overexpressed CK2α (left) and reduced in the tumors that CK2α-silenced (right). **B.** Photographs of dissected tumors from the nude mice. The final tumor volumes were larger in the tumors that overexpressed CK2α (left) and smaller in the tumors that CK2α-silenced (right) than that in the control group. **C.** The tumor weights of each group. The final tumor weights were increased in the tumors that overexpressed CK2α (left) and decreased in the tumors that CK2α-silenced (right). *P*-values were calculated using the independent Student's *t*-test. *P < 0.05 versus control; **P < 0.01 versus control; ***P < 0.001 versus control.

To assess the role of CK2α in tumor growth *in vivo*, the Bel-7402 cells infected with LV-CK2α and LV-NC were injected subcutaneously into nude mice. The results showed that CK2α overexpression in HCC cells significantly promoted tumor growth in the mice (Figure 9A left). The mean tumor volume in the CK2α overexpressed group at the end of observation was significantly larger than that of the control group (1104.86 mm^3^ vs. 226.5 mm^3^). And we also injected the cells that CK2α were knockdown with shCK2α and shNC. Compared with cells transfected shNC, the Bel-7402 cells transfected with shCK2α significantly delayed tumor growth (Figure 9A right). The mean tumor volume in the CK2α knockdown group at the end of observation was significantly smaller than that of the control group (68.42 mm^3^ vs. 345.86 mm^3^). The photographs of dissected tumors from the nude mice were also shown (Figure 9B). Accordingly, the mean tumor weight in the CK2α overexpressed group was markedly higher than that in the control group (0.705 g vs. 0.194 g) (Figure 9C left). And the mean tumor weight in the in the CK2α knockdown group at the end of observation was significantly smaller than that of the control group (0.072 g vs. 0.295 g) (Figure 9C right).

## DISCUSSION

Protein kinase CK2α, one isoform of the catalytic subunit of serine/threonine protein kinase CK2, has been demonstrated to be overexpressed in various malignancies including HCC [23, 32–34]. However, the association between CK2α and clinicopathological features or prognosis for HCC patients remains undefined, and also its biological role in HCC is not defined.

In the present study, we found that CK2α was frequently and significantly up-regulated in human HCC at both the transcriptional (63.8%) and translational (70.9%) levels by RT-qPCR, Western blotting and IHC. Consistent with a recent study by Kim *et al.* [32], our findings convincingly demonstrated that CK2α was overexpressed in primary HCC by IHC analyses. The results showed increased expression of CK2α in 42.9% of HCC samples, and the up-regulated CK2α expression was significantly associated with poorly differentiated HCC, distant metastasis and advanced tumor stage. The relationship between a high expression of CK2α and distant metastasis suggested that the increased expression of CK2α may help accelerate the migration and invasion of tumor cells. These findings indicated that CK2α was an oncogene promoting HCC progression and correlated with pathogenesis.

Importantly, Kaplan-Meier survival analyses revealed that high CK2α expression was significantly correlated with poor overall survival. Furthermore, multivariate Cox regression analyses indicated that CK2α expression was an independent risk factor for overall survival, suggesting that the high expression of CK2α may help in the identification of HCC patients with a poor prognosis. Laramas *et al.* provided evidence for a strong association between aberrant expression of CK2α and poor prognostic factors in human prostate cancer [20]. In addition, studies by Kim *et al.* and Kai-Yuan Lin *et al.* showed that overexpression of CK2α protein in leukemia and colorectal cancer was associated with poor patient outcome [19, 34, 35]. Together with our results, these findings suggest that CK2α may serve as a prognostic marker in various malignancies.

In our study, CK2α was found to have an important role in the biological behavior of HCC. To illustrate the potential regulatory mechanism of CK2α in the development of HCC, a series of functional studies was carried out in HCC cell lines *in vitro*. We altered the expression level of CK2α in hepatoma cells by transfection with targeted siRNAs or CK2α-overexpression vector to investigate its tumor-promoting role in HCC cell lines. CK2α overexpression promoted cell proliferation and colony formation, whereas CK2α silencing inhibited these processes. In tumorigenesis assay, CK2α overexpression in HCC cells significantly promoted tumor growth in the mice. In accord with that result, CK2α-silenced HCC cells by shRNA were significantly delayed tumor growth. These results provide further evidence to confirm CK2α as a candidate oncogene in HCC.

Overexpression of CK2α in human cancers has been associated with angiogenesis and tumor progression [36]. Our additional functional studies also showed that CK2α overexpression increased hepatoma cell migration and invasion. In contrast, silencing CK2α suppressed hepatoma cell motility. These findings were consistent with the results of our clinicopathological analysis, which showed that CK2α overexpression was significantly associated with distant metastasis in the advanced tumor stage. These data showed that abnormal, up-regulated expression of CK2α may promote HCC metastasis. The study by Egeblad *et al.* provided evidence for the involvement of matrix metalloproteinases, which have long been associated with cancer-cell invasion and metastasis, in the association between overexpression of nuclear CK2 and the depth of invasion [37]. Furthermore, Zou *et al.* demonstrated that protein kinase CK2α modulates the cell invasion ability of colorectal cancer cells via regulating epithelial-mesenchymal transition (EMT)-related genes [34].

We also employed cell cycle analyses to monitor changes in the stage of HCC cell division. However, reduced expression of CK2α did not obviously influence the cell cycle distribution compared with control cells. The results suggest that effects of CK2α expression on the HCC cell cycle are minor.

The study conducted by Gray *et al.* indicated that CK2 inhibitor treatment could promote cell apoptosis in breast cancer [38]. Martins *et al.* drew the same conclusion in chronic lymphocytic leukemia [39]. CK2α has been demonstrated to attenuate the apoptosis of human cancers including head and neck squamous cell carcinoma, glioblastoma and prostate cancer [25, 40, 41]. Our studies also found that the inhibition of CK2α expression significantly promoted the apoptosis of HCC cell lines. And we further investigated the regulatory mechanism of CK2α on cell apoptosis. Apoptosis related proteins were detected by western blotting. Strikingly, the effect of CK2α on cell apoptosis has been shown to be achieved through a prominent function in inhibiting that of pro-apoptotic genes *TP53* [25]. In our study, we found total P53 and phosphorylation P53 was markedly up-regulated in CK2α-silenced HCC cells as compared with the control group. Further study showed that inhibited expression of CK2α significantly disrupted the balance of the Bcl-2 family members by decreasing Bcl-2 expression and increasing Bax expression. Kim *et al.* also found the change of Bcl-2 and Bax expression [19]. As a crucial executioner of cell apoptosis, caspase3 and cleaved caspase3 were increased in CK2α-silenced HCC cells. Cleaved caspase9 and cleaved PARP were up-regulated too. These results were consistent with the finding of Turowec *et al.* [42]. PI3K/AKT signaling pathway plays a significantly role in regulating cell survival and apoptosis. Ying Zheng *et al.* found that in accordance with the knockdown of CK2α, the activation of AKT was suppressed [43]. In our study, the *p*-AKT (ser473) was strongly decreased in cells treated with siCK2α. In conclusion, our results indicated that CK2α silencing active apoptosis by regulating the expression of these apoptosis related protein.

In conclusion, our study revealed up-regulated CK2α expression levels in HCC and confirmed the relationship between CK2α overexpression and unfavorable prognosis in HCC patients. CK2α appeared to play an oncogenic role in HCC by promoting tumor-cell growth, colony formation, cell migration, cell invasion and protection against apoptosis. The mouse model experiments revealed that CK2α overexpression significantly promoted the tumor growth. Taken together, our results indicate that CK2α may serve as a candidate prognostic biomarker and a new therapeutic molecular target for HCC.

## MATERIALS AND METHODS

### Patients and tissue samples

A total of 47 pairs of HCC fresh samples and adjacent noncancerous liver tissue samples were collected immediately after surgical tumor resections from primary HCC patients at the Sun Yat-Sen University Cancer Center between 2012 and 2013. None of the patients had undergone transcatheter arterial chemoembolization or chemotherapy before surgery. After surgical resection, fresh tissues were immediately immersed in RNAlater (Ambion, Austin, TX, USA) to avoid RNA degradation and then frozen at −80°C before processing for RNA and protein extraction. An additional 98 paraffin-embedded primary HCC samples, which had been collected between 2001 and 2004, were obtained from patients who had undergone surgery at the Sun Yat-sen University Cancer Center. Serial 2-μm sections from all samples were obtained and used for IHC staining. The histological cell type and stage of tumor tissues were assigned according to the criteria of World Health Organization classification and the tumor node metastasis (TNM) stage set out by the Union for International Cancer Control. Patient post-operative follow-up visits were conducted by our outpatient department. The follow-up included clinical and laboratory examinations (such as serum α-fetoprotein, liver function test and computed tomography) every 3 months in the first 2 years, every 6 months thereafter and then annually for an additional 5 years or until patient death, whichever occurred first. Overall survival, which was used as a measure of prognosis, was defined as the time from surgery to the time of patient's death or the last known follow-up. Before the study, informed consent was obtained from each patient. The study was approved by the Ethics Committee of the Sun Yat-sen University Cancer Center.

### Extraction of total RNA and RT-qPCR

Total RNA was prepared with TRIzol solution (Invitrogen, Shanghai, China). The total RNA concentration and quantity were assessed by absorbency at 260 nm using a Nano Drop spectrophotometer (ND-1000; Thermo Scientific, Wilmington, DE, USA). Reverse transcription was performed using GoScript™ Reverse Transcriptase (Promega, Beijing, China) according to the manufacturer's instructions. The resulting cDNA was then subjected to RT-qPCR for evaluating the relative *CK2α* mRNA expression levels with the reference gene *glyceraldehyde-3-phosphate dehydrogenase* (*GAPDH*) as an internal control. Primers utilized for RT-qPCR were as follows: CK2α forward and reverse primers were 5′-CCGCTTCCACCACAGTTTGA-3′ and 5′-TAAACTCTGGCCCTGCTTGG-3′, respectively; GAPDH forward and reverse primers were 5′-CTCCTCCTGTTCGACAGTCAGC-3′ and 5′-CCCAATACGACCAAATCCGTT-5′, respectively. The RT-qPCR was performed in a final volume of 15 μL in triplicate, consisting of 7.5 μL of 2× SYBR Green master mix (Invitrogen), 2 μL of each 5′ - and 3′ - primer (1.5 pmol/μL), 0.5 μL of sample cDNA and 5 μL of water. The reaction was preheated to 95°C for 10 min, followed by 45 cycles of 95°C for 30 sec and 60°C for 60 sec. Data were analyzed using the comparative threshold cycle (2^−ΔΔCT^) method, and results were averaged and expressed in relative expression units after normalization.

### Protein extraction and western blotting

Western blotting was performed to detect CK2α protein levels in paired clinical specimens from HCC patients and cell lines. Total protein was extracted from freshly frozen tissue samples (tumor tissues and non-tumor control tissues) and cell lines using Radio-Iimmunoprecipitation Assay (RIPA) Lysis Buffer ((Beyotime, Shanghai, China) according to the manufacturer's protocol. The lysates were cleared by centrifugation (12,000 rpm) at 4°C for 30 min, and protein concentrations were measured with a BCA Protein Assay Kit (Thermo Fisher Scientific, Waltham, MA, USA). Briefly, equal amounts of protein (30 μg per sample) were separated by 12% sodium dodecyl sulfatepolyacrylamide gel electrophoresis (SDS-PAGE), electro-transferred onto a polyvinylidene fluoride (PVDF) membrane (Millipore, Billerica, MA, USA) and subsequently blocked with 5% skim milk in TBST for 60 min. The membranes were incubated overnight at 4°C with rabbit polyclonal antibodies against CK2α, P53, *p*-P53(ser15), Bcl-2, Bax (Proteintech, China; 1:1000 dilution), GAPDH (Proteintech; 1:2000 dilution), AKT, *p*-AKT(Ser473), Capase-3, Cleaved Capase-3, Cleaved PARP and Cleaved Capase-9 (Cell Signaling Technology, Danvers, MA, USA; 1:1000 dilution). After three 10-min washes with TBST, the membrane was then incubated with horseradish peroxidase (HRP)-conjugated secondary antibody (Cell Signaling Technology, Danvers, MA, USA; 1:2000 dilution) for 45 min at room temperature. After washing, peroxidase activity was detected on X-ray films using an enhanced chemiluminescence detection system (ECL, Cell Signaling Technology, Danvers, MA, USA). The band intensity was measured by densitometry using Quantity One software (Bio-Rad Laboratories, Hercules, CA, USA). Target protein levels were normalized with respect to GAPDH protein levels.

### IHC and semi-quantitative analysis

Paraffin sections were deparaffinized with dimethylbenzene and rehydrated through 100%, 95%, 90%, 80% and 70% ethanol solutions, followed by three phosphate buffered saline (PBS) washes. For antigen retrieval, slides were boiled in citrate-hydrochloric acid (pH = 6.0) for 15 min in a microwave oven. Endogenous peroxidase activity was blocked in 0.3% hydrogen peroxide at room temperature for 15 min. After rinsing with PBS, non-specific binding was prevented by 5% sheep serum albumin for 30 min. The tissue sections were then incubated with a rabbit polyclonal antibody against CK2α (Millipore; 1:400 dilution) at 4°C overnight. After washing, the sections were incubated for 30 min with HRP-conjugated secondary antibody (Envasion Detection kit; GK500705; Genentech, San Francisco, CA)at room temperature. Following this incubation, the sections were washed three times in PBS, and the visualization signal was developed with 3, 3′-diaminobenzidine tetrahydrochloride (DAB). All of the sections were then counterstained with hematoxylin. The total CK2α immunostaining score was calculated as the sum of the score for proportion of positively stained tumor cells and the score for staining intensity given by two pathologists blinded to the clinical parameters. The proportion of positively stained tumor cells was scored as follows: “0” (<5%, negative), “1” (5% ∼ 25%, sporadic), “2” (25%–50%, focal) and “3” (>50%, diffuse). The intensity of staining was graded according to the following criteria: “0” (no staining); “1” (weak staining = light yellow), “2” (moderate staining = yellow brown) and “3” (strong staining = brown). The total immunostaining score, which ranged from 0 to 9, was calculated as the value of the proportion of positive cells score × staining intensity score. The expression level of CK2α was defined as follows: ‘‘-’’ (negative, score 0), ‘‘+’’ (weakly positive, score 1–3), ‘‘++’’ (positive, score 4–6) or ‘‘+++’’ (strong positive, score 7–9). Thus, CK2α protein expression in HCC specimens was divided into two groups: low CK2α expression group (CK2α“–” or CK2α“+”) and high CK2α expression group (CK2α“++” or CK2α“+++”).

### Cell lines and cell cultures

The human HCC cell lines Hep3B and HepG2 and a human liver adenocarcinoma endothelial cell line SK-Hep1 were obtained from the American Type Culture Collection (Manassas, VA, USA). The SMMC-7721 cell line was obtained from the Chinese Academy of Science (Shanghai, China). The Huh7 cell line was obtained from the Health Science Research Resources Bank (Osaka, Japan). The Bel-7402 cell line and the normal liver cell line LO2 were obtained from the Committee of Type Culture Collection of the Chinese Academy of Sciences (Shanghai, China).

### RNA oligonucleotides and cell transfections

Small interfering RNAs (siRNAs) were synthesized by GenePharma (Suzhou, China). For our transfection analyses, 2 × 10^5^ cells were seeded in 6-well plates and transfected with siRNA. The four CK2α siRNA (siCK2α) sequences were as follows: siCK2α#1, sense, 5′-GUGGAUUUAUAGUAGUUCATT-3′ and antisense 5′-UGAACUACUAUAAAUCCACTT-3′; siCK2α#2, sense, 5′-CCUCCCAAAUUUAGUUCCUTT-3′ and antisense 5′-AGGAACUAAAUUUGGGAGGTT-3′; siCK2α#3, sense, 5′-CCUAAAUCCAACUCAUUUATT-3 and antisense 5′-UAAAUGAGUUGGAUUUAGGTT-3′; siCK2α#4, sense, 5′-CCCUUGCUGUGUGUAUAU ATT-3′ and antisense 5′-UAUAUACACACAGC AAGGGTT-3′. For the negative control siRNA (siNC): sense, 5′-UUCUCCGAACGUGUCACGUTT-3′ and antisense, 5′-ACGUGACACGUUCGGAGAATT-3′. The four CK2α siRNAs were transfected into cells using Lipofectamine RNAiMax reagent (Invitrogen) according to manufacturer's instruction. Two different siRNAs, siCK2α#1 and siCK2α#3, effectively knocked down the amount of CK2α in the transfected cells. And plasmid-vector for short hairpin RNAs (shRNAs) for knockout of CK2α (shCK2α) and negative control (shNC) were also synthesized by GenePharma (Suzhou, China). Bel-7402 cells were transfected with the indicated shRNAs using RNAi-Mate (Suzhou, China) according to manufacturer's protocol. After infection for 48 h, the cells were selected in the presence of 3 μg/mL Geneticin (G418, Sigma, St. Louis, MO) and G418-resistant cells were pooled and cultured for further analysis. The stable cell lines were designated as shCK2α and shNC, respectively. Knockdown efficiency was evaluated by Western blotting.

Recombinant lentiviruses overexpressing CK2α (LV-CK2α) and negative control vector (LV-NC) were obtained from GenePharma (Suzhou, China). Lentiviral infection was performed by adding virus solution to HepG2 and Bel-7402 cells in the presence of 5 μg/mL polybrene (Sigma-Aldrich, St. Louis, MO). After infection for 48 h, the cells were selected in the presence of 3 μg/mL puromycin, and puromycin-resistant cells were pooled and cultured for further analysis. The stable cell lines were designated as HepG2-LV-CK2α, HepG2-LV-NC, Bel-7402-LV-CK2α, and Bel-7402-LV-NC, respectively.

### Proliferation assay

A (3-(4,5-dimethylthiazol-2-yl)-5-(3-carboxymethoxyphenyl)-2-(4-sulfophenyl)-2H-tetrazolium) (MTS) assay (Sigma-Aldrich, St Louis, MO, USA) was used to measure the growth rates of cells. The cells which were collected after transfection with indicated siRNAs and recombinant lentiviruses were plated into 96-well plates in triplicate at 1 × 10^3^ per well. After 24 h, 20 μL of MTS (5 mg/ml) was added to cells for quantifying cell proliferation from 1 to 7 days. The cells were incubated with MTS for 3 h in 5% CO_2_ at 37°C. Finally, optical absorbance of each well was measured at 490 nm using a microplate reader. Cell growth curves were made by plotting the absorbance (ordinate) against time (abscissa). Three independent experiments were performed to analyze the cell growth.

### Colony formation assay

For analysis of cell colony formation, transfected cells were routinely harvested, resuspended in complete medium and then placed in 6-well plates (1,000 cells per well). Three control wells were seeded with the same number of cells as the experimental wells. After 10 days of conventional incubation, the surviving colonies were fixed and stained with crystal violet. Colonies which contained 50 or more cells were counted. Colony-forming efficiency (CFE, %) was calculated using the formula: CFE = (colony number/plated cell number) × 100. The experiments were carried out three times independently.

### Cell cycle assay

For the cell cycle assay, transfected cells were routinely collected and centrifuged after 48 h. Total cells were washed twice with PBS and fixed with 75% ethanol at −20°C overnight. The cells were then washed in cold PBS, resuspended in 400 μL PBS containing 20 μL RNaseA and incubated in 37°C for 30 min. Propidium iodide (PI; Bestbio, Shanghai, China) was used to stain cells at 4°C in the dark for 45 min. The cellular DNA content was quantified using a flow cytometer (Beckman Coulter, Brea, CA, USA). All experiments were performed three times.

### Apoptosis assay

For the apoptosis assay, cells were routinely collected and centrifuged after transfection. After washing with cold PBS twice, cells were resuspended in 400 μL 1× binding buffer and then incubated with 5 μL Annexin V-FITC (Bestbio) and 10 μL PI for 15 min in the dark at 4°C. Stained cell numbers were analyzed by flow cytometry (Beckman Coulter). All experiments were performed three times.

### Cell migration assay

Cell migration assays were carried out using a chamber system consisting of polycarbonate membrane inserts with an 8-μm pore size (Corning, Corning, NY, USA) placed in 24-well cell culture insert companion plates. Cells (5 × 10^4^) in 200 μL RPMI 1640 containing 5% fetal bovine serum (FBS) were seeded in the upper chamber, and 600 μL RPMI 1640 containing 15% FBS was placed in the lower chamber at 48 h. After incubation at 37°C for 24 h, the cells remaining in the upper chamber were removed with cotton swabs. The insert membranes were then fixed with 75% methanol for 30 min, stained with 0.5% crystal violet for 60 min and counted. The stained cells in 10 random microscopic fields per membrane were counted. Each experiment was performed in triplicate.

### Matrigel invasion assay

Matrigel invasion assays were carried out using a chamber system consisting of polycarbonate membrane inserts with an 8-μm pore size (Corning) placed in 24-well cell culture insert companion plates. The inserts were coated with a thin layer of 0.5 mg/ml Matrigel Basement Membrane Matrix (BD Biosciences, Bedford, MA, USA). Briefly, transfected cells were resuspended in RPMI 1640 containing 5% FBS. Cells (4 × 10^5^) in 200 μL of growth medium were added to the upper chamber, and the lower chamber was filled with 600 μL of growth medium containing 15% FBS. After incubation at 37°C for 48 h, non-migrating cells were removed from the upper chamber with a cotton swab. Invading cells on the bottom of the filter were fixed with 75% methanol for 30 min, stained with 0.5% crystal violet for 60 min and counted. The stained cells in 10 random microscopic fields per membrane were counted. Each experiment was conducted in triplicate.

### Tumorigenicity assays in nude mice

Femal balb/c athymic nude mice (4 ∼ 5 weeks old) were obtained from the Medical Experiment Animal Center of Guandong Province. The mice were randomly assigned to 4 groups (*n* = 7) before inoculation. Group 1 was injected with Bel-7402 cells that have been injected with LV-CK2α; Group 2 was injected with Bel-7402 cells that have been injected with LV-NC. Group 3 and Group 4 were injected with Bel-7402 cells that have been injected with shCK2α and shNC. For the injection, 2 × 10^6^ tumor cells were suspended in 100 μL PBS including 30% Matrigel Basement Membrane Matrix (BD Biosciences, Bedford, MA, USA). And then the cells were subcutaneously injected into the right axilla of the mice. The tumor size was monitored every 3 days by measuring the length (L) and width (W) of the tumor with calipers. The tumor volume was calculated according the following formula: (L ×W^2^)/2. At 4–5 weeks after inoculation, all the mice were sacrificed and the tumor were harvested and photographed. The weight of tumors was also measured. All the experimental procedures involving animals were performed in accordance with the Guide for the Care and Use of Laboratory Animals (NIH publications Nos. 80–23. revised 1996) and the institutional ethical guidelines for animal experiments.

### Statistical analysis

All statistical analyses were carried out with the SPSS statistical software package (version 16.0; SPSS, Inc., Chicago, IL, USA). Survival curves were calculated by Kaplan-Meier analysis and compared using the log-rank test. Correlations between CK2α expression and the clinical variables were analyzed using the Pearson χ^2^ test. Comparisons between groups were analyzed using the Student *t*-test, unless otherwise specified. In addition, a Cox proportional hazards regression model was used to identify factors that were independently associated with overall survival. All tests were two-sided, and *P* < 0.05 was considered statistically significant.

## References

[1] Altekruse SF, McGlynn KA, Reichman ME (2009). Hepatocellular carcinoma incidence, mortality, and survival trends in the United States from 1975 to 2005. Journal of clinical oncology : official journal of the American Society of Clinical Oncology.

[2] El-Serag HB (2012). Epidemiology of Viral Hepatitis and Hepatocellular Carcinoma. Gastroenterology.

[3] Thomas MB, Jaffe D, Choti MM, Belghiti J, Curley S, Fong Y, Gores G, Kerlan R, Merle P, O'Neil B, Poon R, Schwartz L, Tepper J, Yao F, Haller D, Mooney M (2010). Hepatocellular carcinoma: consensus recommendations of the National Cancer Institute Clinical Trials Planning Meeting. Journal of clinical oncology : official journal of the American Society of Clinical Oncology.

[4] Thomas MB, Zhu AX (2005). Hepatocellular carcinoma: the need for progress. Journal of clinical oncology : official journal of the American Society of Clinical Oncology.

[5] Njei B, Rotman Y, Ditah I, Lim JK (2015). Emerging trends in hepatocellular carcinoma incidence and mortality. Hepatology.

[6] Nakakura EK, Choti MA (2000). Management of hepatocellular carcinoma. Oncology (Williston Park, NY).

[7] El-Serag HB, Marrero JA, Rudolph L, Reddy KR (2008). Diagnosis and treatment of hepatocellular carcinoma. Gastroenterology.

[8] Bruix J, Sherman M (2011). Management of hepatocellular carcinoma: An update. Hepatology.

[9] Guerra B, Issinger OG (2008). Protein kinase CK2 in human diseases. Current medicinal chemistry.

[10] Litchfield DW (2003). Protein kinase CK2: structure, regulation and role in cellular decisions of life and death. The Biochemical journal.

[11] Pinna LA (2002). Protein kinase CK2: a challenge to canons. Journal of cell science.

[12] Tawfic S, Yu S, Wang H, Faust R, Davis A, Ahmed K (2001). Protein kinase CK2 signal in neoplasia. Histology and histopathology.

[13] Ahmad KA, Wang G, Unger G, Slaton J, Ahmed K (2008). Protein kinase CK2 - A key suppressor of apoptosis. Advances in Enzyme Regulation.

[14] Ahmed K, Gerber DA, Cochet C (2002). Joining the cell survival squad: an emerging role for protein kinase CK2. Trends in cell biology.

[15] Moucadel V, Prudent R, Sautel CF, Teillet F, Barette C, Lafanechere L, Receveur-Brechot V, Cochet C (2011). Antitumoral activity of allosteric inhibitors of protein kinase CK2. Oncotarget.

[16] Dominguez I, Sonenshein GE, Seldin DC (2009). Protein kinase CK2 in health and disease: CK2 and its role in Wnt and NF-kappaB signaling: linking development and cancer. Cellular and molecular life sciences : CMLS.

[17] Bae JS, Park SH, Kim KM, Kwon KS, Kim CY, Lee HK, Park BH, Park HS, Lee H, Moon WS, Chung MJ, Sylvester KG, Jang KY (2015). CK2alpha phosphorylates DBC1 and is involved in the progression of gastric carcinoma and predicts poor survival of gastric carcinoma patients. International journal of cancer Journal international du cancer.

[18] Duncan JS, Litchfield DW (2008). Too much of a good thing: the role of protein kinase CK2 in tumorigenesis and prospects for therapeutic inhibition of CK2. Biochimica et biophysica acta.

[19] Kim JS, Eom JI, Cheong JW, Choi AJ, Lee JK, Yang WI, Min YH (2007). Protein kinase CK2alpha as an unfavorable prognostic marker and novel therapeutic target in acute myeloid leukemia. Clinical cancer research : an official journal of the American Association for Cancer Research.

[20] Laramas M, Pasquier D, Filhol O, Ringeisen F, Descotes JL, Cochet C (2007). Nuclear localization of protein kinase CK2 catalytic subunit (CK2alpha) is associated with poor prognostic factors in human prostate cancer. European journal of cancer.

[21] Lin KY, Fang CL, Chen Y, Li CF, Chen SH, Kuo CY, Tai C, Uen YH (2010). Overexpression of nuclear protein kinase CK2 Beta subunit and prognosis in human gastric carcinoma. Annals of surgical oncology.

[22] Martins LR, Lucio P, Silva MC, Anderes KL, Gameiro P, Silva MG, Barata JT (2010). Targeting CK2 overexpression and hyperactivation as a novel therapeutic tool in chronic lymphocytic leukemia. Blood.

[23] Zhang S, Wang Y, Mao JH, Hsieh D, Kim IJ, Hu LM, Xu Z, Long H, Jablons DM, You L (2012). Inhibition of CK2alpha down-regulates Hedgehog/Gli signaling leading to a reduction of a stem-like side population in human lung cancer cells. PloS one.

[24] Pizzi M, Piazza F, Agostinelli C, Fuligni F, Benvenuti P, Mandato E, Casellato A, Rugge M, Semenzato G, Pileri SA (2015). Protein kinase CK2 is widely expressed in follicular, Burkitt and diffuse large B-cell lymphomas and propels malignant B-cell growth. Oncotarget.

[25] Brown MS, Diallo OT, Hu M, Ehsanian R, Yang X, Arun P, Lu H, Korman V, Unger G, Ahmed K, Van Waes C, Chen Z (2010). CK2 modulation of NF-kappaB, TP53, and the malignant phenotype in head and neck cancer by anti-CK2 oligonucleotides *in vitro* or *in vivo* via sub-50-nm nanocapsules. Clinical cancer research : an official journal of the American Association for Cancer Research.

[26] Gu L, Husain-Ponnampalam R, Hoffmann-Benning S, Henry RW (2007). The protein kinase CK2 phosphorylates SNAP190 to negatively regulate SNAPC DNA binding and human U6 transcription by RNA polymerase III. The Journal of biological chemistry.

[27] Canton DA, Litchfield DW (2006). The shape of things to come: an emerging role for protein kinase CK2 in the regulation of cell morphology and the cytoskeleton. Cellular signalling.

[28] Guo C, Davis AT, Yu S, Tawfic S, Ahmed K (1999). Role of protein kinase CK2 in phosphorylation nucleosomal proteins in relation to transcriptional activity. Molecular and cellular biochemistry.

[29] Broker LE, Kruyt FA, Giaccone G (2005). Cell death independent of caspases: a review. Clinical cancer research : an official journal of the American Association for Cancer Research.

[30] el-Deiry WS (1998). Regulation of p53 downstream genes. Seminars in cancer biology.

[31] Zhang L, Zhou F, ten Dijke P (2013). Signaling interplay between transforming growth factor-beta receptor and PI3K/AKT pathways in cancer. Trends in biochemical sciences.

[32] Kim HS, Chang YG, Bae HJ, Eun JW, Shen Q, Park SJ, Shin WC, Lee EK, Park S, Ahn YM, Park WS, Lee JY, Nam SW (2014). Oncogenic potential of CK2alpha and its regulatory role in EGF-induced HDAC2 expression in human liver cancer. The FEBS journal.

[33] Shimada K, Anai S, Marco DA, Fujimoto K, Konishi N (2011). Cyclooxygenase 2-dependent and independent activation of Akt through casein kinase 2alpha contributes to human bladder cancer cell survival. BMC urology.

[34] Zou J, Luo H, Zeng Q, Dong Z, Wu D, Liu L (2011). Protein kinase CK2alpha is overexpressed in colorectal cancer and modulates cell proliferation and invasion via regulating EMT-related genes. Journal of translational medicine.

[35] Lin KY, Tai C, Hsu JC, Li CF, Fang CL, Lai HC, Hseu YC, Lin YF, Uen YH (2011). Overexpression of nuclear protein kinase CK2 alpha catalytic subunit (CK2alpha) as a poor prognosticator in human colorectal cancer. PloS one.

[36] Trembley JH, Chen Z, Unger G, Slaton J, Kren BT, Van Waes C, Ahmed K (2010). Emergence of protein kinase CK2 as a key target in cancer therapy. BioFactors.

[37] Egeblad M, Werb Z (2002). New functions for the matrix metalloproteinases in cancer progression. Nature Reviews Cancer.

[38] Gray GK, McFarland BC, Rowse AL, Gibson SA, Benveniste EN (2014). Therapeutic CK2 inhibition attenuates diverse prosurvival signaling cascades and decreases cell viability in human breast cancer cells. Oncotarget.

[39] Martins LR, Perera Y, Lucio P, Silva MG, Perea SE, Barata JT (2014). Targeting chronic lymphocytic leukemia using CIGB-300, a clinical-stage CK2-specific cell-permeable peptide inhibitor. Oncotarget.

[40] Dixit D, Sharma V, Ghosh S, Mehta VS, Sen E (2012). Inhibition of Casein kinase-2 induces p53-dependent cell cycle arrest and sensitizes glioblastoma cells to tumor necrosis factor (TNFalpha)-induced apoptosis through SIRT1 inhibition. Cell death & disease.

[41] Hessenauer A, Schneider CC, Gotz C, Montenarh M (2011). CK2 inhibition induces apoptosis via the ER stress response. Cellular signalling.

[42] Turowec JP, Vilk G, Gabriel M, Litchfield DW (2013). Characterizing the convergence of protein kinase CK2 and caspase-3 reveals isoform-specific phosphorylation of caspase-3 by CK2alpha’: implications for pathological roles of CK2 in promoting cancer cell survival. Oncotarget.

[43] Zheng Y, McFarland BC, Drygin D, Yu H, Bellis SL, Kim H, Bredel M, Benveniste EN (2013). Targeting protein kinase CK2 suppresses prosurvival signaling pathways and growth of glioblastoma. Clinical cancer research : an official journal of the American Association for Cancer Research.

